# Bacterial vaginosis: a review of approaches to treatment and prevention

**DOI:** 10.3389/frph.2023.1100029

**Published:** 2023-05-31

**Authors:** Carmen Abbe, Caroline M. Mitchell

**Affiliations:** ^1^ Elson S. Floyd College of Medicine, Washington State University, Spokane, WA, United States; ^2^ Vincent Center for Reproductive Biology, Massachusetts General Hospital, Boston, MA, United States

**Keywords:** bacterial vaginosis, vaginitis, gardnerella vaginalis, emerging therapies, vaginal microbiome

## Abstract

Bacterial vaginosis (BV) is a common cause of vaginitis worldwide and is associated with serious reproductive health outcomes, including increased risk of preterm birth, sexually transmitted infections, and pelvic inflammatory disease. The current and only FDA-approved treatment regimens for BV are antibiotics, such as metronidazole and clindamycin. Antibiotics provide a short-term cure for bacterial vaginosis; however, fail to provide a consistent long-term cure for many women. Fifty to eighty percent of women experience a BV recurrence within a year of completing antibiotic treatment. This may be because after antibiotic treatment, beneficial strains of *Lactobacillus*, such as *L. crispatus,* do not recolonize the vagina. In the absence of an effective long-term cure, patients, providers, and researchers are exploring different approaches to treatment and prevention, resulting in a rapid evolution of perspectives on BV pathogenesis and approaches to management. Current areas of investigation for BV management include probiotics, vaginal microbiome transplantation, pH modulation, and biofilm disruption. Behavioral modifications that may help include smoking cessation, condom use and hormonal contraception. Additional strategies considered by many people include dietary modification, non-medical vaginally applied products, choice of lubricant, and treatments from medical practices outside of allopathic medicine. This review aims to provide a comprehensive and up to date outline of the landscape of ongoing and potential treatment and prevention strategies for BV.

## 1. Introduction

Bacterial vaginosis (BV) is the most common cause of vaginitis among reproductive aged women, commonly presenting with vaginal discharge and odor (1). BV is characterized by a decline in abundance of *Lactobacillus,* a healthy vaginal bacteria (2, 3), and a simultaneous overgrowth of pathogenic bacteria, such as *Gardnerella vaginalis, Atopobium vaginae, Megasphaera spp., Prevotella spp. and Sneathia spp*. Point-of-care diagnosis of BV can be made by noting at least three of four Amsel criteria, which include thin, white discharge, clue cells on microscopy, a vaginal pH > 4.5, and a fishy odor with application of potassium hydroxide (4). Laboratory based molecular tests, which identify DNA from a subset of BV-associated species, have recently been FDA approved (5). Research studies often use the Nugent score of bacterial morphotypes on vaginal fluid Gram stain for diagnosis (6).

In the United States, the estimated prevalence of BV is 29%–49% (7, 8). Despite this high prevalence, an effective long-term cure for BV remains elusive for many women. There is a high recurrence rate of BV following antibiotics (9–11), the only approved therapeutic regimen, and many women struggle with recurrent BV [defined as ≥3 episodes in a 12 month period (11)]. Such high recurrence may be due to persistence of the infection, re-exposure to BV-associated organisms, or a failure of lactobacilli to recolonize the vagina. Lack of adequate treatment for BV is a significant public health issue both because of the impact of symptoms on quality of life, and because women with BV are at increased risk for preterm birth (12, 13), sexually transmitted infections (STIs) including HIV(14, 15), and pelvic inflammatory disease(16). Additionally, women with BV are at increased risk for post-operative complications such as vaginal cuff cellulitis (17).

Various studies have found racial differences in BV prevalence (18). It is important to highlight that race is not a biologic factor, suggesting that there are environmental or situational factors linked to the lived experience of a racial or ethnic group, including racism (19), that may contribute to differences in BV prevalence. While differences in BV prevalence may exist across race or ethnicity (20), there is no known difference in treatment efficacy.

The failure of antibiotics to provide a long-term cure for BV has led many women and clinicians to consider alternative therapies. The focus of this review is to discuss the evidence for potential non-antibiotic therapeutic and preventive options for BV, including those currently under investigation and those more informally used (Table 1).

**Table 1:** Overview of interventions for treatment and/or prevention of bacterial vaginosis.

Intervention	Proposed mechanism of action	Example	Clinical evidence
Antibiotics	Antibacterial agents that act via inhibition of bacterial protein synthesis	Metronidazole, Clindamycin (22)	High quality evidence of benefit
Vaginal microbiome transplant (VMT)	Introduction of an exogenous bacterial microbiome to help restore eubiosis	Vaginal microbiome transplantation (23)	Low quality evidence for benefit
pH modulation	Modulation of vaginal pH to become more acidic, promoting an environment in which BV associated bacteria grow poorly	Lactic acid (24) Vitamin C (25)	Moderate quality evidence for no effect
Biofilm disruption	Disruption of the biofilms that may exist on BV associate bacteria, increasing the bacteria's susceptibility to antibacterial mechanisms	Boric acid (26) Astodrimer vaginal gel (27)	Moderate quality evidence for benefit
Probiotics	Recolonization of the vagina with health-promoting *Lactobacillus* species	Oral (28) and vaginally administered probiotics (25, 29–32), such as *L. crispatus* (33, 34)*.*	Low to Moderate quality evidence for benefit
Diet	Modulation of the body's immune response to bacterial pathogenesis and/or interplay between the gastrointestinal and vaginal microbiomes	Avoidance of sugar (35, 36) and fat (37) Intake of Vitamins A, C, E, D (38)	Low to Moderate quality evidence for benefit
Barrier Contraception	Preventing the introduction of bacteria to the vaginal environment	Condom use (39)	Moderate quality evidence for benefit
Hormonal contraception	Immunoregulation by estrogen and progesterone and/or decreased menses	Oral contraceptive pill, hormonal IUD (40)	Moderate quality evidence for benefit
Smoking Cessation	Alteration of vaginal microbiota and potential increase in biogenic amines	Cessation of smoking (41)	Low to moderate quality evidence for benefit

Strength of clinical evidence determined through use of GRADE criteria (21), with specific factors driving classification of a body of evidence outlined in Supplementary Table S1.

## 2. The current treatment paradigm

Antibiotics are the first line treatment for BV. The recommended therapeutic regimens include oral or intravaginal metronidazole and intravaginal clindamycin. These treatments have similar efficacy and are effective for short-term resolution of the infection (22). Recurrent bacterial vaginosis is a common drawback to current treatment options. Within 6–12 months of finishing antibiotic therapy, 50%–80% of women will experience a bacterial vaginosis recurrence (9–11). Proposed reasons for this treatment failure include reinfection via sexual partners, antimicrobial resistance, biofilm, and failure to reestablish a health-optimal vaginal microbiota. A more comprehensive review of the nuances of current antibiotic treatment regimens can be found in two review papers by Muzny et al. (42, 43).

There is a debate in the field as to whether BV can be transmitted sexually via male or female partners; however, studies have shown that treating male partners with antibiotics does not affect bacterial vaginosis reoccurrence in female partners (44–46). No studies have evaluated treatment of female partners in female-female partnerships.

Antibiotic resistance and biofilm persistence are other proposed reasons for long-term treatment failure. One study found that out of 50 strains of *Gardnerella vaginalis,* the majority were resistant to both metronidazole and clindamycin (47). Another study found increased antimicrobial resistance to clindamycin among anaerobic isolates following previous treatment (48). Although there is a paucity of data, a recent review discusses the studies that have attempted to examine antibiotic resistance in BV (49). In an analysis of available *in vivo* data, they indicate there is increasingly convincing evidence of antimicrobial resistance in BV and a need to address this in clinical management (49). Persistent polymicrobial biofilm, which has been more often identified in people with recurrent BV compared to healthy people or those with a single episode, may play a part in antimicrobial resistance (50). Bacterial biofilm reduces antimicrobial penetrance and even after clinically successful bacterial vaginosis antibiotic therapy, biofilm persists (51).

Failure of antimicrobial *Lactobacillus* to recolonize the vagina following antibiotic therapy may be another reason for high BV recurrence. Among the *Lactobacillus* species most commonly found in the vagina, *Lactobacillus crispatus* is most correlated with a stable, lactobacilli-rich vaginal microbiome, while *Lactobacillus iners* correlates with vaginal microbiome instability (52, 53). Although antibiotic therapy reduces the quantity of *Gardnerella vaginalis* and other BV-associated species, the post-antibiotic treatment microbiota are usually dominated by *L. iners* rather than the species considered more beneficial (*L. crispatus, L. jensenii)* (54, 55). The failure to restore a non-*iners Lactobacillus* rich microbial community is a possible reason that antibiotics have failed to provide a long-term cure to recurrent BV.

## 3. Probiotics

Probiotics are live microorganisms that can be ingested through diet or supplements, or can be administered vaginally. Probiotics containing *Lactobacillus* are often used and marketed for the management of BV and may be beneficial in preventing recurrent BV through recolonization of the vaginal microbiota. *Lactobacillus* species have been shown to have antimicrobial effects against BV-associated species (53, 56, 57). Their use has been studied as both a treatment for BV as well as to prevent recurrence of BV after antibiotic therapy.

Studies of probiotics vary widely in species included in the product, study methodology, and final outcomes. The majority of studies used species of *Lactobacillus* that are prominent in the gut, not vagina (58). A wide variety of *Lactobacillus* strains exist, and their abundance differs between the gut and vagina (59, 60). *Lactobacillus crispatus, L. iners, L. gasseri* and *L. jensenii* are the species most commonly found in the vaginal tract (60, 61). Even among these strains, there is variability, with *L. crispatus* associated with a protective effect on vaginal health while *L. iners* is associated with a more unstable vaginal microbiome (52, 53). Despite this wide variability in environmental abundance and effect of strains, the majority of oral probiotics marketed for vaginal health contain *Lactobacillus* strains that are more commonly found in the gut than vagina (58), and very few contain *L. crispatus*.

The mechanism of action of oral probiotics, which are more commonly used, is unknown. It is possible that oral probiotics may reach the vaginal microbiome through the gastrointestinal tract. Two studies have demonstrated that bacterial species may be shared between intestinal and vaginal tracts (62, 63) and a cross-sectional study found that co-colonization of the rectum and vagina by hydrogen peroxide-producing lactobacilli was associated with lower prevalence of BV (63). A randomized controlled trial (*N* = 544) found that people with BV taking oral probiotics experienced a higher rate of restitution to *Lactobacillus*-rich vaginal microbiota at six weeks than in the placebo group (61.5% vs. 26.9%) (28). This research did not compare the effect of oral probiotics to existing antibiotic therapies. A comprehensive systemic review assessed randomized control trials using oral and vaginal probiotics for BV treatment (64). They identified four trials meeting their inclusion criteria and found no conclusive evidence that probiotics are comparable to or enhance the effectiveness of antibiotics. They did report a suggestible beneficial outcome of combined use of probiotics and antibiotics. Three out of the four identified studies used vaginal, rather than oral, probiotics.

Unlike oral probiotics, vaginal probiotics have the advantage of direct administration. Many trials are underpowered to determine whether probiotics are equivalent to existing therapies. Even among five randomized controlled trials that include more than 80 participants (and thus, approach reasonable power), results vary. Two placebo-controlled studies comparing antibiotic therapy and antibiotics plus vaginal probiotics found comparable cure rates (29) and recurrence (30) of BV between the two arms. Another study (*N* = 100) found that, when administered following antibiotic therapy, vaginal probiotics did not significantly impact BV cure rate, but they did prolong time without BV recurrence (64.9% vs. 46.2%) (31). Similar results were found in a study (*N* = 115) examining the effect of probiotics vs. antibiotic treatment; the probiotic arm had a higher 30-day cure rate than the antibiotic arm (96% vs. 70%), and among clinically cured participants, the probiotic arm had a lower BV recurrence rate (32). Finally, a randomized controlled trial (*N* = 190) demonstrated that vaginal probiotics significantly improved vaginal microbiota restoration following antibiotic treatment (25)—an outcome associated with decreased risk for BV recurrence. Even among these large-scale randomized controlled trials, the effectiveness of probiotics remains inconclusive. This is likely due to the variability of *Lactobacillus* species used across studies.

Two randomized studies have examined the effect of *L. crispatus* on BV recurrence after antibiotic treatment. In a phase III randomized clinical trial (*N* = 98), women with recurrent BV previously treated with metronidazole were given two treatments of vaginally administered *L. crispatus,* also known as Physioflor (34). The administration of *L. crispatus* significantly reduced the incidence of BV recurrence (20%) compared to those in the placebo group (41%). Of note, this study had a considerable amount of missing data, with only 78 participants' data included in their analysis, which introduces significant bias. A more robust, phase IIb randomized, placebo-controlled study examined the role of an intravaginally applied *L. crispatus* live biotherapeutic product, also known as Lactin-V, in managing recurrent BV among 228 women (33). Lactin-V was administered to women within 48 h of finishing a course of metronidazole, daily for 5 days and then twice weekly for 10 weeks. They found that at 12 weeks, women administered Lactin-V had a significantly lower incidence of BV recurrence (30%) than those who received placebo (45%). A secondary outcome demonstrated that at 24 weeks after antibiotic treatment, *L. crispatus* was detected in 48% of women who had received Lactin-V and 2% of women who received placebo. This well conducted study demonstrated that among women with recurrent BV, the vaginal administration of *L. crispatus* significantly reduces BV recurrence (33).

There is insufficient evidence to recommend commercially available probiotics at this time.

## 4. Vaginal microbiome transplantation

Vaginal microbiome transplantation (VMT) is a novel therapeutic option currently under investigation for prevention of recurrent BV. In VMT, women receive vaginal fluid collected from healthy donors. Similar to fecal microbiome transplantation, which can successfully treat recurrent *C. difficile* infections (65), VMT introduces an exogenous bacterial community to help restore eubiosis. Unlike probiotics, which introduce isolated species of bacteria into the vaginal microbiota, VMT transfers a whole microbiome.

A 2019 case series was the first to assess the effectiveness of VMT (23). The study enrolled five women with recurrent BV who had previously undergone multiple antibiotic regimens. Following an intravaginal antibiotic treatment, the women received transplanted vaginal fluids from donors. Two out of the five women had full remission of BV after one transplant, and two more after multiple transplants, for the duration of follow up (5–21 months) with no adverse effects. The fifth woman had partial remission. This study demonstrated that VMT can change the composition of vaginal microbiota. The four women for whom VMT was successful had vaginal microbiota dominated by *L. crispatus* for the duration of follow up. *L. gasseri* dominated the microbiota of the patient with unsuccessful VMT. This finding supports the hypothesis that *L. crispatus* may be the most beneficial species of *Lactobacillus* for restoring healthy vaginal microbiota.

This is a promising outcome, suggestive that VMT has potential to restore healthy vaginal microbiota and treat recurrent BV. However, there was no control group and some of the women required multiple VMTs to achieve full remission. Additionally, donors must be screened appropriately and thoroughly to minimize potential risks to recipients. Further research is needed before this approach can be adopted clinically. At the time of this review, per clinicaltrials.gov there are two ongoing randomized trials of VMT, one in Israel and one in the United States. This procedure should not be performed outside of clinical trials.

## 5. pH modulation

Lactobacilli produce lactic acid, which helps maintain a low, acidic, vaginal pH (66). BV-associated anaerobic bacteria grow poorly in acidic environments, which has led many to hypothesize that lactic acid could provide protective effects against vaginal infections (67). BV is characterized by loss of lactobacilli and the lactic acid they produce, leading to an increase in vaginal pH, which may allow the anaerobic overgrowth that is the hallmark of this syndrome (68, 69). The administration of lactic acid, and other pH modulators, therefore, may hold potential in the management of BV (2).

Studies of pH modulating agents including lactic acid, mucoadhesive vaginal gel, and acetic acid in women with BV have mostly been small, with sample sizes of less than 100. Results from these studies have varied: effects range from no benefit (70, 71) to providing a clinical cure (72, 73). A literature review concluded that lactic-acid containing agents do not significantly affect vaginal microbiota (74). A randomized controlled trial (*N* = 409) which compared lactic acid gel to oral metronidazole for clinical cure of BV found that lactic acid was less effective than metronidazole at resolving BV symptoms (47% vs. 70%) in a two-week span. For those in the study whose symptoms cleared in the initial 2 weeks, both lactic acid and metronidazole had correspondingly high recurrence rates at 70% and 71% (24).

Vaginally administered vitamin C has also been demonstrated to decrease vaginal pH (75–77). When used after antibiotic treatment, vitamin C helps maintain an acidic vaginal environment, allowing more time for restoration of healthy vaginal microbiota (75). A randomized controlled trial (*N* = 277) demonstrated that vitamin C, applied for 6 days intravaginally, had a higher clinical cure rate for BV than placebo (44% vs. 22%) (78). A subsequent randomized controlled trial (*N* = 142) found that intravaginal vitamin C significantly decreased BV recurrence rate for up to 6 months compared to placebo (16% vs. 32%) (79). However, in this trial, there was no difference in vaginal pH between people who did vs. did not receive vaginal Vitamin C during treatment, suggesting that any mechanism of effect was not related to lowering of pH. Of note, the formulation used in these studies is a silicone-coated tablet that slowly releases the ascorbic acid, which is not available in the United States.

There is no reliable evidence that pH modulators alone are effective at treating BV or that they are more effective than existing antibiotic treatments. In vitro experiments continue to provide biologic plausibility for elevated pH as a permissive mechanism to allow growth of BV-associated organisms, but it is not yet clear whether a pH altering product can counter that *in vivo*. Thus, pH modulating products should not be recommended at this time.

## 6. Biofilm disruptors

One hypothesized reason for high rates of recurrence of BV is the presence of a polymicrobial biofilm (80–85). Biofilms are composed of microbial cells and an extracellular matrix that can provide bacterial protection, and are generally associated with decreased efficacy of antimicrobial agents (86). Fluorescence in-situ hybridization (FISH) studies using vaginal biopsies demonstrate an adherent layer of bacteria in many people with BV (84). However, there is still some debate about whether this is a true biofilm, as no studies have yet demonstrated the presence of an extracellular matrix.

There are a variety of agents that are purported to target biofilms. *Thymbra capitata,* an essential oil, has antimicrobial effects on *Gardnerella* spp. (87) and BV-associated polymicrobial biofilms *in vitro* (88). Although not yet studied *in vivo*, it may hold promise in BV management. Dequalinium chloride (DQC) is a quaternary ammonium compound that has antimicrobial activity against vaginal pathogens such as *Gardnerella* (89, 90) and it can partially disrupt *in vitro Gardnerella* biofilms on plastic culture plates (91). A randomized non-inferiority trial (*N* = 321) demonstrated DQC can also successfully treat BV clinically, with cure rates similar to vaginally administered clindamycin 25 days after treatment (74.8%vs. 74.8%) (92), however it is not available commercially in the United States.

PM-477, a genetically engineered endolysin, is asserted to destroy *Gardnerella* biofilms *in vitro*. Preclinical data demonstrates that PM-477 effectively inhibits *Gardnerella* biofilm growth and that it is not prone to resistance development, unlike metronidazole and clindamycin (93). PM-477 may also be effective at disrupting polymicrobial biofilms which include organisms other than *Gardnerella* (94). This agent has not yet been studied *in vivo*.

Boric acid is a chemical that, while not FDA-approved, is commonly used by women attempting to manage persistent BV (26). In vitro it inhibits biofilm formation by *Staphylococcus aureus* and *Pseudomonas auruginosa* (95). However, it did not decrease the viability of S. aureus in an existing biofilm (96). A retrospective chart review examined clinical use of boric acid for recurrent vulvovaginal candidiasis and BV in 272 patients. They found that long term use of boric acid was well tolerated, had high reported satisfaction by patients, and few adverse effects (97). A recent study (*N* = 105) found that boric acid, used in conjunction with antibiotic therapies, resulted in a 69% six month cure for women with recurrent BV (98). A randomized trial of two formulations of TOL-463, a boric-acid based therapy, in 106 participants demonstrated 50%–59% efficacy in early clinical cure (9–12 days) of BV (99). These results are promising and, if studied in the setting of recurrent BV, may have potential in clinical management. Despite its relatively widespread use in BV management, there is limited research on boric acid in the treatment of vaginitis and no studies have examined boric acid alone in the setting of recurrent BV. Additionally, boric acid is used as a pesticide, and although the EPA has found that it is not a carcinogen, the long-term safety of its topical use in humans is unexplored (100).

Astodrimer Sodium, also referred to as Astodrimer 1% Vaginal gel, is a polyanionic dendrimer that prevents formation of bacterial biofilms through blocking bacterial adhesion (27). Randomized, placebo-controlled studies have demonstrated greater clinical cure rates for BV compared to placebo (46.2% vs. 11.5%) (101) and comparable to current antibiotic treatments (102). A recent randomized control trial (*N* = 864) demonstrated that Astodrimer, when applied every other day for 16 weeks following antibiotic therapy, was associated with a 20% reduction in recurrent BV compared to placebo during 16-week follow up (27). This product is not commercially available in the United States, but is sold over the counter in the United Kingdom (Betafem), Europe (Betadine BV) and also in Australia, New Zealand, Southeast Asia and South Africa.

Of the products discussed, Astodrimer and dequalinium chloride have the most evidence of benefit in BV, however neither are available in the United States.

## 7. Smoking

**Figure 1:**
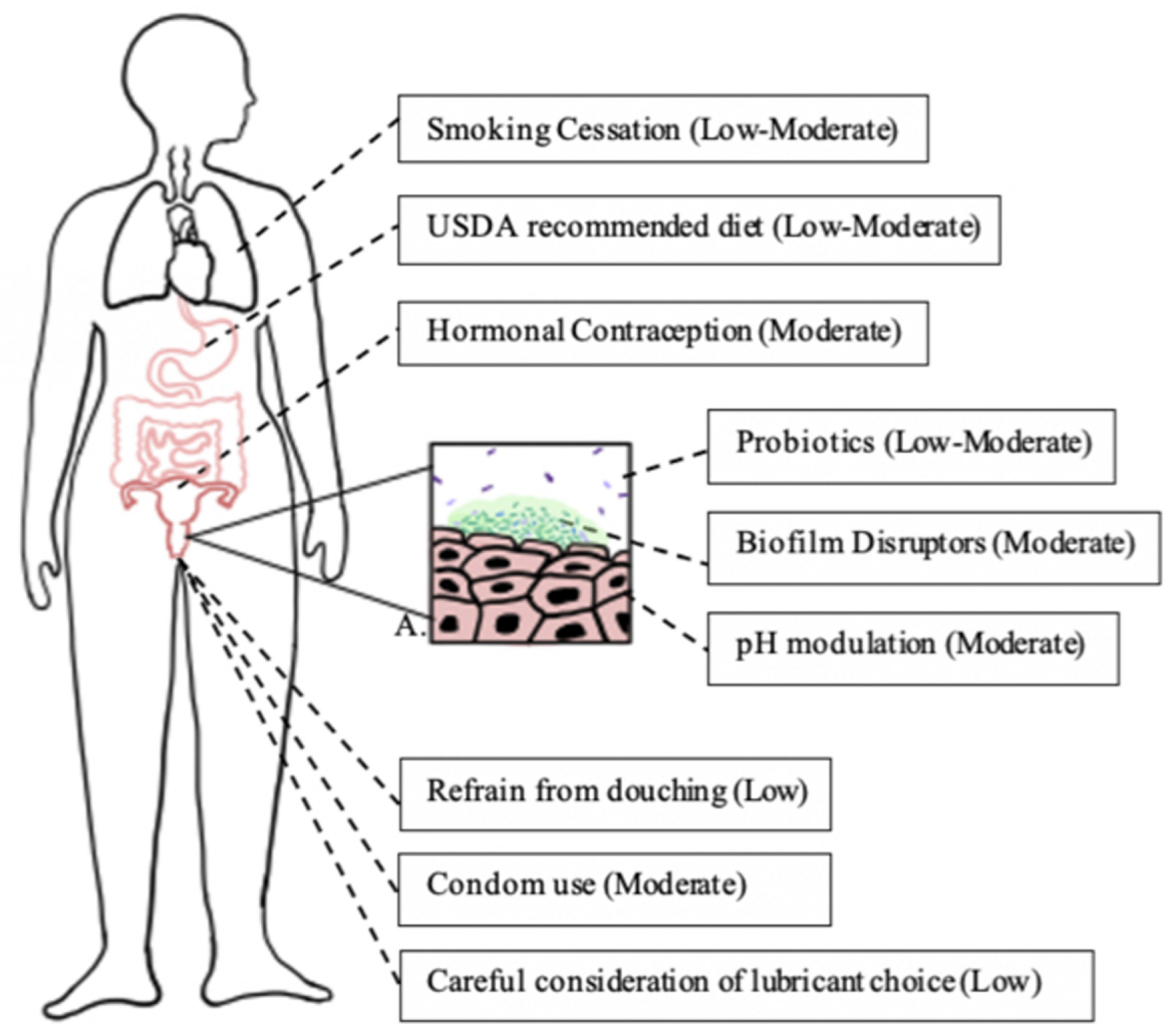
Lifestyle changes associated with reduced risk of bacterial vaginosis. Each box represents a lifestyle modification that may affect vaginal epithelium and bacterial vaginosis risk and the modification's quality of evidence, as determined by the GRADE criteria (108). Box A represents a bacterial vaginosis-pathogen dominated vaginal epithelium (dark green rods), with development of biofilm (light green) and reduction of lactobacilli (purple rods).

It is well known that smoking has adverse effects on the body, including increased risk for infections (103). A cohort study of 956 women found smoking to be a significant risk factor for BV (adjusted odds ratio of 3.0) (41) and various other studies have found smoking to be an independent factor related to BV incidence (7, 104, 105). A small cross sectional study (*N* = 20) found that vaginal microbial communities of smokers were more likely to be *Lactobacillus* depleted while nonsmokers were more likely to have *L. crispatus* dominated microbiota (106). Another study identified that women who smoke have higher concentrations of biogenic amines, which may promote non-*Lactobacillus* species and increase vaginal pH (107). Smoking is a modifiable risk factor of BV and is an important lifestyle change to consider in its management (Figure 1).

## 8. Contraception

Both hormonal and non-hormonal contraceptives may play a role in BV occurrence. A large scale cross sectional study (*N* = 16,314) found that BV is more common in women using the non-hormonal copper intrauterine device (IUD) (14.8%) than women with hormonal IUDs (9.7%) and women who are not IUD users (11.1%) (109). Another large study (*N* = 2,585), as a secondary analysis, found that copper IUDs increased women's risk of BV 1.28 fold compared to other non-hormonal methods (110). A well-powered systematic review and meta-analysis found that hormonal contraceptive use, regardless of type, is associated with reduced prevalence, incidence, and recurrence of BV (40).

Various studies have examined condom use in relation to BV occurrence and prevalence. A systematic review and meta-analysis found that condom use was protective against BV, with an estimated relative risk of 0.8 (39). A case-crossover study among 871 women found that consistent condom use was protective against BV (111). Existing research suggests that copper IUDs may increase risk of BV, while condoms and hormonal forms of contraception may decrease risk of BV (Figure 1).

Contraceptive choice is very individual, however if a person with recurrent BV is interested in a hormonal contraceptive method rather than a copper IUD, this may reduce the risk for BV. Additionally, using condoms—at least during antibiotic therapy for BV—may be beneficial.

## 9. Complementary and alternative options

In today's health care climate, many now turn to the internet for answers to their medical questions. Online forums and articles describe several “home remedies” to cure BV. In the following section we discuss several remedies often mentioned online (Table 2). Unfortunately, there are few rigorous studies on these therapies, making it difficult to draw conclusions about their potential efficacy.

**Table 2:** Overview of complementary and alternative options for the management and prevention of bacterial vaginosis.

Intervention	Study type and population	Outcome
**Diet**		
Fat intake	Longitudinal study, *N* = 1,521 (37)	Increased total fat intake is associated with increased risk of BV; OR = 1.4–2.3
Folate	Longitudinal study, *N* = 1,521 (37)	Increased folate is associated with decreased risk of BV; OR = 0.40
Vitamin E	Longitudinal study, *N* = 1,521 (37)	Increased vitamin E is associated with decreased risk of BV; OR = 0.41
Calcium	Longitudinal study, *N* = 1,521 (37)	Increased calcium is associated with decreased risk of BV; OR = 0.40
Glycemic load	Case control study, *N* = 144 (36) Cohort study, *N* = 1,735 (35)	Dietary glycemic index and glycemic load are associated with increased risk and prevalence of BV**;** OR = 1.04–4.01
Fiber	Case control study, *N* = 144 (36) Cross sectional study, *N* = 104 (112)	Diets rich in fiber correlate with decreased risk of BV; OR = 0.22; 0.49
Beta-carotene	Cross sectional analysis, *N* = 553 (38)	Higher levels beta-carotene correlate with decreased risk of BV; OR = 9.2
Vitamin D	RCT, *N* = 118 (113)	Vitamin D intake does not affect risk of BV
Yogurt & other fermented foods	No *in vivo* studies.	No *in vivo* studies
Apple cider vinegar	No *in vivo* studies.	No *in vivo* studies
Garlic	Single-blinded RCT (without adherence to CONSORT guidelines), *N* = 120 (114)	Garlic and metronidazole have similar effectiveness in BV treatment
Lubricant	Cohort, *N* = 44 (115)	Lubricant use is correlated with few differences in vaginal inflammation, with a trend toward decreased *L. crispatus*
**Essential Oils**		
Thymbra capita	In vitro study (88) No *in vivo* studies.	Thymbra capita oil has antimicrobial effects on BV biofilm
Tea Tree	In vitro study (116) No *in vivo* studies.	BV pathogens have susceptibility to tea tree oil
Traditional Chinese Medicine	Case study, *N* = 180 (117)	*Cortex Phellodendri Chinesis* was associated with improvement of BV symptoms and Nugent score
**Douching**		
	Large cross sectional study, *N* = 1,200 (118)	Vaginal douching is associated with BV and BV-related microbiota; OR = 2.1.
	Cross sectional study, *N* = 609 (119)	Vaginal douching is associated with lactobacilli-depleted microbiomes; OR = 2.24
	Cross sectional study, *N* = 272 (120)	Vaginal douching is associated with reduction of beneficial lactobacilli; prevalence ratio = 0.57
	Cross sectional study, *N* = 234 (121)	Association between vaginal washing and BV-associated bacteria is variable geographically
	Longitudinal Observational study, *N* = 100 (122)	Douching is associated with reduced clearance of BV; OR = 0.45
	Pilot study, *N* = 38 (123)	Cessation of vaginal douching may reduce risk of BV; OR = 0.76

The adjusted odds ratio (aOR) reported by each study is included when available.

### 9.1. Diet

While diet is a significant predictor of gut microbiota composition, it is not yet understood what role diet plays in determining vaginal microbial communities. It is possible that changes in diet may affect the vaginal microbiota through modulating the body's immune response to bacterial pathogenesis and/or through the interplay between the gastrointestinal and vaginal microbiomes (62, 63). A handful of cross-sectional and case control studies conducted mostly in the United States have examined how diet impacts the vaginal microbiome and vaginitis. These studies demonstrate correlations between BV and high glycemic load (35, 36), high dietary fat (37), low dietary fiber (36, 112), and poor intake of Vitamins (A, C, E, D) (38) and micronutrients (38, 124).

A large longitudinal study across over 1500 women in Alabama found that high total fat intake was a significant predictor of BV (37). They also found that high intake of folate, vitamin E and calcium were significantly associated with decreased risk of severe BV (Nugent score ≥9 and vaginal pH ≥5), with adjusted odds ratios of 0.40, 0.41, and 0.40, respectively (37). A separate study among 553 women in the United States found a significant correlation between deficiency in the micronutrient β-carotene and increased risk of BV, with an adjusted odds ratios of 9.2 (38). β-carotene is a micronutrient commonly found in fruits and vegetables and is a precursor to Vitamin A. Deficiencies in the micronutrient betaine, found in seafood and spinach, may also correlate with incident BV (124). Two recent studies also demonstrated that diets rich in fiber are significantly correlated with decreased risk of BV, with odds ratios of 0.22 (36) and 0.49 (112).

The oral intake of garlic, which has known antimicrobial properties, has also been explored as an antibacterial agent for BV management. Allicin, a compound from garlic, has previously been demonstrated to have antifungal properties (125); however, these antifungal properties do not directly translate to antibacterial properties that might target BV. Garlic's antibacterial properties have been sparsely studied in the context of BV. One single-blinded study (*n* = 120) found that oral garlic tablets and oral metronidazole have similar effectiveness in treating BV (114); however, the study was underpowered and did not adhere to CONSORT guidelines for reporting. No other studies have examined garlic intake in the context of BV.

Many fermented foods are rich in *Lactobacillus* strains. The ingestion of fermented foods, such as yogurt or kimchi, function similarly to oral probiotics via the introduction of live bacteria to the gastrointestinal system. The effect of oral probiotic consumption on the management of BV is still not well understood but may have potential, as discussed in the probiotics section. Whether it is more effective to deliver live bacteria in food vs. as a capsule has not been evaluated.

Vitamin D, which is acquired from both dietary sources and UV-B, may play a role in BV occurrence. Researchers have examined its effects with conflicting results. Multiple studies among pregnant women have found that Vitamin D deficiency correlates with increased BV risk (126–128); however, a large scale longitudinal study (*N* = 2,337) did not find a significant relationship between Vitamin D deficiency and BV among non-pregnant women (129). Additionally, a randomized trial of Vitamin D supplementation, which demonstrated a significant increase in serum vitamin D in treated participants, did not demonstrate a significant reduction in BV incidence vs. placebo (65% vs. 48%) (113).

Dietary carbohydrate indices, such as glycemic load, can be useful indicators for carbohydrate rich diets that result in increased postprandial serum glucose. Women with diabetes are at greater risk of vaginal dysbiosis (130) and vaginal infections (131). A case control study among Iranian women (*N* = 295) found that high dietary glycemic index and glycemic load were significantly associated with increased risk of BV, with adjusted odds ratios of 2.99 and 4.01 (36). A cohort study of 1735 women found glycemic load to be associated with slightly greater incidence and persistence of BV, with odds ratios of 1.03 and 1.02 (35).

Available research seems to suggest that a diet which follows the USDA recommendations reduces risk of BV. Many studied micronutrients interact with one another in the body, making it difficult to confidently separate their effects. The extent to which diet could play a part in the clinical management of BV is not known, although current data provide a foundation upon which future research could build.

### 9.2. Apple cider vinegar

Online articles discuss apple cider vinegar baths, douches, and oral consumption in the context of BV management. A 2018 *in vitro* study demonstrated that apple cider vinegar has antimicrobial effect on pathogens such as *S. aureus* and *E. coli* (132), but its effects on BV-associated bacteria have not been studied. Apple cider vinegar contains acetic acid and lactic acid, both of which have been studied in the context of BV management. One proposed mechanism of action of these properties is through acidification of the vaginal pH, making it less hospitable to BV-associated species that thrive at a higher pH. As we discuss in the pH modulation section, simply modifying the vaginal pH may not be sufficient to prevent BV. Oral consumption of apple cider vinegar would likely not have a direct effect on the vagina but may influence gut microbiota.

### 9.3. Lubricants

The World Health Organization (WHO) recommends that lubricants do not exceed an 1,200 mOsm/kg osmolality, which may be associated with vaginal cytotoxicity (133, 134), and recommends that vaginal lubricants have a pH near 4.5 (135). These guidelines are based on promoting vaginal epithelial integrity and decreasing risk for sexually transmitted infections. Otherwise, little is known regarding the effect of lubricant on BV. Various online forums discuss how the use of lubricant during intercourse may help prevent BV recurrence.

A recent *in vitro* study found that different vaginal products have varying effects on growth of beneficial *L. crispatus*. They found that two vaginal lubricants inhibited growth of *L. crispatus,* while another stimulated its growth (136). An *in vivo* study (*N* = 44) examining vaginal microbiota after lubricant use and non-use found few significant changes in vaginal inflammatory markers between the groups, as well as a trend towards decreased *L. crispatus* in the women who used lubricant (115); however, this study did not control for type of lubricant used. Depending on the product, lubricant may increase vaginal epithelial inflammation and dysbiosis (137, 138), affect vaginal microbiota through antimicrobial preservatives such as parabens or glycerine (139), and/or alter microbiota through pH and osmolality (139). The effect lubricants have on vaginal microbiome and BV are likely product specific. Further, products that induce mucosal inflammation, even if they have beneficial effects for prevention of BV, may increase the risk for acquisition of other STIs.

### 9.4. Douching

Vaginal douching is a method of flushing a product through the vagina, often as an attempt to address vaginal discomfort or discharge. An *in vitro* 2021 study found that the contents of common commercial douching products (vinegar, iodine and baking soda) all may be associated with epithelial inflammation and disruption of the anti-inflammatory effects of lactobacilli (140). It is hypothesized that increased inflammation may inhibit recolonization of beneficial lactobacilli. Douching has been associated with reduction of *L. crispatus*, with no reduction of *L. iners* or *L. jensenii* (120)*.* An early pilot study among 39 women found that cessation of vaginal douching may reduce BV risk (123). A recent longitudinal observational study (*N* = 100) found that among women with *L. iners* dominated vaginal microbiota, douching was related with reduced clearance of BV (122). A cross sectional study among 1,200 women found that douching is associated with BV and BV-related pathogens (118). An international cross-sectional study (*N* = 234) found an associated between douching and BV-associated bacteria in American women but not among Kenyan women (121). Finally, a cross-sectional study of 609 women found that vaginal douching, regardless of product, is associated with lactobacilli-depleted microbiomes (119). However, most *in vivo* douching studies have been cross-sectional, limiting the ability to draw causal inference. Additionally, many people douche because of symptoms of BV, thus associations between douching and lower counts of lactobacilli may not be causally related. However, because of the possible association with decreased vaginal lactobacilli, and its association with pelvic inflammatory disease, douching should be discouraged (141).

### 9.5. Traditional Chinese medicine

Traditional Chinese medicine (TCM) uses therapeutic herbs in the prevention and treatment of disease, including mediation of microbiota (142). There are various approaches to treating BV with TCM (143), which include utilizing the immunity-enhancing and antimicrobial properties of medicinal herbs. Much of the clinical research on TCM in the context of BV have small sample sizes, and many of the research publications, completed in China, are not widely available on U.S. research databases. One available study examines berberine, the active ingredient of the herbal medicine *Cortex Phellodendri Chinesis*. A study of 240 Chinese women found that clinical symptoms of women in the BV arm (*N* = 180) significantly improved following topical treatment with berberine and the Nugent score improved in 92.78% (117). There was no control BV group, which makes these results difficult to analyze. Much of the clinical research on TCM in BV and vaginal health management has been done in the last few years and has recently been compiled into a published review (143). More clinical research is needed to better understand its potential effect on BV management.

### 9.6. Essential oils

In the 1990s, early research indicated that tea tree oil may have potential to manage BV symptoms (116, 144). A 1999 *in vitro* study, found that BV-associated bacteria are susceptible to tea tree oil while lactobacilli are more resistant (116). While these publications are referenced online, they are not peer reviewed nor do they have large sample sizes. Anecdotally, many providers report that patients have vaginal irritation with the use of tea tree oil.

Thymbra capita essential oil has also been studied in the context of BV management. Thymbra capita, a type of thyme, has well-established antimicrobial properties (108, 145, 146). Recently, a study on *in vitro* and ex vivo vaginal tissue models demonstrated that the oil's antimicrobial properties extend to *Gardnerella,* while sparing healthy *Lactobacillus* species (87). A subsequent study examining BV biofilm *in vitro* found that the biofilm model, containing six BV-associated species, was susceptible to the antibacterial effects of thymbra capita oil (117), suggesting that it may be effective against a variety of BV-associated pathogens. In this study, thymbra capita significantly reduced the total mass of the polymicrobial biofilm and posed no harm to vaginal epithelial cells. Notably, bacterial species present in this study were resistant to metronidazole, suggesting this essential oil could be a viable option for recurrent BV resistant to antibiotics. However, no data are available on how well tolerated this product would be, nor the impact on vaginal epithelium.

Additional BV management methods that are widely discussed online, such as vitamin C, boric acid, and oral probiotics, are discussed in previous sections of this article.

## 10. Conclusion

Bacterial vaginosis is a common medical diagnosis that significantly impacts women's quality of life and reproductive health. Antibiotics, the only approved treatment for BV, have high recurrence rates, negative side effects, and after antibiotic treatment, there is limited recolonization of the vaginal microbiota with beneficial *Lactobacillus* species. Despite high prevalence of BV worldwide and the inability of antibiotics to provide a long-term cure, few effective alternative treatment options exist. There are various studies underway attempting to identify novel approaches, with possible solutions ranging from diet and lifestyle changes to biofilm disruption, pH modulation and vaginal microbiome transplantation. Still, much of this research is either underpowered or concentrated on *in vitro* models. Considering the lack of science-based answers, clinicians and patients alike are attempting to find solutions on their own. It is important that medical research continues to prioritize BV management and that both clinicians and patients have the resources to be informed on the safety and efficacy of the products that many are already using.

## References

[1] Nasioudis D Linhares IM Ledger WJ Witkin SS. Bacterial vaginosis: a critical analysis of current knowledge. BJOG. (2017) 124(1):61–9. 10.1111/1471-0528.14209 27396541

[2] O’Hanlon DE Moench TR Cone RA. Vaginal pH and microbicidal lactic acid when lactobacilli dominate the microbiota. PLoS One. (2013) 8(11):e80074. 10.1371/journal.pone.0080074 PMC3819307 24223212

[3] Stoyancheva G Marzotto M Dellaglio F Torriani S. Bacteriocin production and gene sequencing analysis from vaginal Lactobacillus strains. Arch Microbiol. (2014) 196(9):645–53. 10.1007/s00203-014-1003-1 24919535

[4] Colonna C Steelman M. Amsel Criteria. StatPearls.( 2021). Available at: https://www.ncbi.nlm.nih.gov/books/NBK542319/ (Cited July 2, 2022). 31194459

[5] Gaydos CA Beqaj S Schwebke JR Lebed J Smith B Davis TE Clinical validation of a test for the diagnosis of vaginitis. Obstet Gynecol. (2017) 130(1):181–9. 10.1097/AOG.0000000000002090 28594779 PMC5635603

[6] Nugent RP Krohn MA Hillier SL. Reliability of diagnosing bacterial vaginosis is improved by a standardized method of gram stain interpretation. J Clin Microbiol. (1991) 29(2):297. 10.1128/jcm.29.2.297-301.1991 1706728 PMC269757

[7] Koumans EH Sternberg M Bruce C McQuillan G Kendrick J Sutton M The prevalence of bacterial vaginosis in the United States, 2001−2004; associations with symptoms, sexual behaviors, and reproductive health. Sex Transm Dis. (2007) 34(11):864–9. 10.1097/OLQ.0b013e318074e565 17621244

[8] Bitew A Abebaw Y Bekele D Mihret A. Prevalence of bacterial vaginosis and associated risk factors among women complaining of genital tract infection. Int J Microbiol. (2017) 2017:Article ID 4919404. 10.1155/2017/4919404 28831285 PMC5558670

[9] Bradshaw CS Morton AN Hocking J Garland SM Morris MB Moss LM High recurrence rates of bacterial vaginosis over the course of 12 months after oral metronidazole therapy and factors associated with recurrence. J Infect Dis. (2006) 193(11):1478–86. 10.1086/503780 16652274

[10] Coudray MS Madhivanan P. Bacterial vaginosis—a brief synopsis of the literature. Eur J Obstet Gynecol Reprod Biol. (2020) 245:143–8. 10.1016/j.ejogrb.2019.12.035 31901667 PMC6989391

[11] Faught BM Reyes S. Characterization and treatment of recurrent bacterial vaginosis. J Womens Health (Larchmt). (2019) 28(9):1218–26. 10.1089/jwh.2018.7383 31403349

[12] Hay PE Lamont RF Taylor-Robinson D Morgan DJ Ison C Pearson J. Abnormal bacterial colonisation of the genital tract and subsequent preterm delivery and late miscarriage. Br Med J. (1994) 308(6924):295. 10.1136/bmj.308.6924.295 8124116 PMC2539287

[13] Hillier SL Nugent RP Eschenbach DA Krohn MA Gibbs RS Martin DH Association between bacterial vaginosis and preterm delivery of a low-birth-weight infant. The vaginal infections and prematurity study group. N Engl J Med. (1995) 333(26):1737–42. 10.1056/NEJM199512283332604 7491137

[14] Martin HL Richardson BA Nyange PM Lavreys L Hillier SL Chohan B Vaginal lactobacilli, microbial flora, and risk of human immunodeficiency virus type 1 and sexually transmitted disease acquisition. J Infect Dis. (1999) 180(6):1863–8. 10.1086/315127 10558942

[15] Atashili J Poole C Ndumbe PM Adimora AA Smith JS. Bacterial vaginosis and HIV acquisition: a meta-analysis of published studies. AIDS. (2008) 22(12):1493–501. 10.1097/QAD.0b013e3283021a37 18614873 PMC2788489

[16] Wiesenfeld HC Hillier SL Krohn MA Amortegui AJ Heine RP Landers DV Lower genital tract infection and endometritis: insight into subclinical pelvic inflammatory disease. Obstet Gynecol. (2002) 100(3):456–63. 10.1016/s0029-7844(02)02118-x 12220764

[17] Soper DE. Bacterial vaginosis and surgical site infections. Am J Obstet Gynecol. (2020) 222(3):219–23. 10.1016/j.ajog.2019.09.002 31499057

[18] Peebles K Velloza J Balkus JE McClelland RS Barnabas RV. High global burden and costs of bacterial vaginosis: a systematic review and meta-analysis. Sex Transm Dis. (2019) 46(5):304–11. 10.1097/OLQ.0000000000000972 30624309

[19] Ukoha EP Snavely ME Hahn MU Steinauer JE Bryant AS. Toward the elimination of race-based medicine: replace race with racism as preeclampsia risk factor. Am J Obstet Gynecol. (2022) 227(4):593–6. 10.1016/j.ajog.2022.05.048 35640703

[20] Fettweis JM Paul Brooks J Serrano MG Sheth NU Girerd PH Edwards DJ Differences in vaginal microbiome in African American women versus women of European ancestry. Microbiology. (2014) 160(Pt 10):2272. 10.1099/mic.0.081034-0 25073854 PMC4178329

[21] What is GRADE?. BMJ Best Pract. (2022). Available from: https://bestpractice.bmj.com/info/us/toolkit/learn-ebm/what-is-grade/

[22] Oduyebo OO Anorlu RI Ogunsola FT. The effects of antimicrobial therapy on bacterial vaginosis in non-pregnant women. Cochrane Database Syst Rev. (2009) (3):CD006055. 10.1002/14651858.CD006055.pub2 19588379

[23] Lev-Sagie A Goldman-Wohl D Cohen Y Dori-Bachash M Leshem A Mor U Vaginal microbiome transplantation in women with intractable bacterial vaginosis. Nat Med. (2019) 25(10):1500–4. 10.1038/s41591-019-0600-6 31591599

[24] Armstrong-Buisseret L Brittain C Kai J David M Anstey Watkins J Ozolins M Lactic acid gel versus metronidazole for recurrent bacterial vaginosis in women aged 16 years and over: the VITA RCT. Health Technol Assess. (2022) 26(2):1–170. 10.3310/ZZKH4176 35057905

[25] Petricevic L Witt A. The role of Lactobacillus casei rhamnosus Lcr35 in restoring the normal vaginal flora after antibiotic treatment of bacterial vaginosis. Available from: www.blackwellpublishing.com/bjog (Cited January 23, 2022). 10.1111/j.1471-0528.2008.01882.x 18823487

[26] Workowski KA Bachmann LH Chan PA Johnston CM Muzny CA Park I Sexually transmitted infections treatment guidelines, 2021. MMWR Recomm Reports. (2021) 70(4):1–187. 10.15585/mmwr.rr7004a1 PMC8344968 34292926

[27] Schwebke JR Carter BA Waldbaum AS Agnew KJ Paull JRA Price CF A phase 3, randomized, controlled trial of Astodrimer 1% Gel for preventing recurrent bacterial vaginosis. Eur J Obstet Gynecol Reprod Biol X. (2021) 10:100121. 10.1016/j.eurox.2021.100121 33537666 PMC7843408

[28] Vujic G Jajac Knez A Despot Stefanovic V Kuzmic Vrbanovic V. Efficacy of orally applied probiotic capsules for bacterial vaginosis and other vaginal infections: a double-blind, randomized, placebo-controlled study. Eur J Obstet Gynecol Reprod Biol. (2013) 168(1):75–9. 10.1016/j.ejogrb.2012.12.031 23395559

[29] Eriksson K Carlsson B Forsum U Larsson P-G. A Double-blind Treatment Study of Bacterial Vaginosis with Normal Vaginal Lactobacilli after an Open Treatment with Vaginal Clindamycin Ovules. 10.1080/00015550410022249 15848990

[30] Bradshaw CS Pirotta M de Guingand D Hocking JS Morton AN Garland SM Efficacy of oral metronidazole with vaginal clindamycin or vaginal probiotic for bacterial vaginosis: randomised placebo-controlled double-blind trial. PLoS One. (2012) 7(4):e34540. 10.1371/journal.pone.0034540 PMC3317998 22509319

[31] Larsson PG Stray-Pedersen B Ryttig KR Larsen S. Human lactobacilli as supplementation of clindamycin to patients with bacterial vaginosis reduce the recurrence rate; a 6-month, double-blind, randomized, placebo-controlled study. BMC Womens Health. (2008) 8(1):1–8. 10.1186/1472-6874-8-3 18197974 PMC3225869

[32] Ling Z Liu X Chen W Luo Y Yuan L Xia Y The restoration of the vaginal microbiota after treatment for bacterial vaginosis with metronidazole or probiotics. Microb Ecol. (2013) 65(3):773–80. 10.1007/s00248-012-0154-3 23250116

[33] Cohen CR Wierzbicki MR French AL Morris S Newmann S Reno H Randomized trial of lactin-V to prevent recurrence of bacterial vaginosis. N Engl J Med. (2020) 382(20):1906–15. 10.1056/NEJMoa1915254 32402161 PMC7362958

[34] Bohbot JM Daraï E Bretelle F Brami G Daniel C Cardot JM. Efficacy and safety of vaginally administered lyophilized Lactobacillus crispatus IP 174178 in the prevention of bacterial vaginosis recurrence. J Gynecol Obstet Hum Reprod. (2018) 47(2):81–6. 10.1016/j.jogoh.2017.11.005 29196153

[35] Thoma ME Klebanoff MA Rovner AJ Nansel TR Neggers Y Andrews WW Bacterial vaginosis is associated with variation in dietary indices. J Nutr. (2011) 141(9):1698. 10.3945/jn.111.140541 21734062 PMC3159055

[36] Noormohammadi M Eslamian G Kazemi SN Rashidkhani B Malek S. Association of dietary glycemic Index, glycemic load, insulin Index, and insulin load with bacterial vaginosis in Iranian women: a case-control study. Infect Dis Obstet Gynecol. (2022) 2022:e1225544. 10.1155/2022/1225544 35370395 PMC8970957

[37] Neggers YH Nansel TR Andrews WW Schwebke JR Yu KF Goldenberg RL Dietary intake of selected nutrients affects bacterial vaginosis in women. J Nutr. (2007) 137(9):2128. 10.1093/jn/137.9.2128 17709453 PMC2663425

[38] Tohill BC Heilig CM Klein RS Rompalo A Cu-Uvin S Piwoz EG Nutritional biomarkers associated with gynecological conditions among US women with or at risk of HIV infection. Am J Clin Nutr. (2007) 85(5):1327–34. 10.1093/ajcn/85.5.1327 17490970

[39] Fethers KA Fairley CK Hocking JS Gurrin LC Bradshaw CS. Sexual risk factors and bacterial vaginosis: a systematic review and meta-analysis. Clin Infect Dis. (2008) 47(11):1426–35. 10.1086/592974 18947329

[40] Vodstrcil LA Hocking JS Law M Walker S Tabrizi SN Fairley CK Hormonal contraception is associated with a reduced risk of bacterial vaginosis: a systematic review and meta-analysis. PLoS One. (2013) 8(9):73055. 10.1371/journal.pone.0073055 PMC3762860 24023807

[41] Hellberg D Nilsson S Mårdh PA. Bacterial vaginosis and smoking. Int J STD AIDS. (2000) 11(9):603–6. 10.1258/0956462001916461 10997505

[42] Muzny CA Kardas P. A narrative review of current challenges in the diagnosis and management of bacterial vaginosis. Sex Transm Dis. (2020) 47(7):441–6. 10.1097/OLQ.0000000000001178 32235174 PMC7294746

[43] Muzny CA Balkus J Mitchell C Sobel JD Workowski K Marrazzo J Diagnosis and management of bacterial vaginosis : summary of evidence reviewed for the 2021 centers for disease control and prevention sexually transmitted infections treatment guidelines. Clin Infect Dis. (2022) 74(Suppl 2):S144–S151. 10.1093/cid/ciac021 35416968

[44] Swedberg J Steiner JF Deiss F Steiner S Driggers DA. Comparison of single-dose vs one-week course of metronidazole for symptomatic bacterial vaginosis. JAMA. (1985) 254(8):1046–9. 10.1001/jama.1985.03360080058029 3894707

[45] Colli E Landoni M Parazzini F. Treatment of male partners and recurrence of bacterial vaginosis: a randomised trial. Genitourin Med. (1997) 73(4):267. 10.1136/sti.73.4.267 9389947 PMC1195855

[46] Moi H. Prevalence of bacterial vaginosis and its association with genital infections, inflammation, and contraceptive methods in women attending sexually transmitted disease and primary health clinics. Int J STD AIDS. (1990) 1(2):86–94. 10.1177/095646249000100203 1965491

[47] Nagaraja P. Antibiotic resistance of gardnerella vaginalis in recurrent bacterial vaginosis. Indian J Med Microbiol. (2008) 26(2):155–7. 10.4103/0255-0857.40531 18445953

[48] Beigi RH Austin MN Meyn LA Krohn MA Hillier SL. Antimicrobial resistance associated with the treatment of bacterial vaginosis. Am J Obstet Gynecol. (2004) 191(4):1124–9. 10.1016/j.ajog.2004.05.033 15507930

[49] Muzny CA Sobel JD. The role of antimicrobial resistance in refractory and recurrent bacterial vaginosis and current recommendations for treatment. Antibiotics. (2022) 11(4):500. 10.3390/antibiotics11040500 35453251 PMC9024683

[50] Swidsinski A Mendling W Loening-Baucke V Ladhoff A Swidsinski S Hale LP Adherent biofilms in bacterial vaginosis. Obstet Gynecol. (2005) 106(5 Pt 1):1013–23. 10.1097/01.AOG.0000183594.45524.d2 16260520

[51] Swidsinski A Loening-Baucke V Swidsinski S Verstraelen H. Polymicrobial Gardnerella biofilm resists repeated intravaginal antiseptic treatment in a subset of women with bacterial vaginosis: a preliminary report. Arch Gynecol Obstet. (2015) 291(3):605–9. 10.1007/s00404-014-3484-1 25245669

[52] Brotman RM Shardell MD Gajer P Tracy JK Zenilman JM Ravel J Interplay between the temporal dynamics of the vaginal Microbiota and human papillomavirus detection. J Infect Dis. (2014) 210(11):1723. 10.1093/infdis/jiu330 24943724 PMC4296189

[53] Verstraelen H Verhelst R Claeys G De Backer E Temmerman M Vaneechoutte M. Longitudinal analysis of the vaginal microflora in pregnancy suggests that L. crispatus promotes the stability of the normal vaginal microflora and that L. gasseri and/or L. iners are more conducive to the occurrence of abnormal vaginal microflora. BMC Microbiol. (2009) 9:116. 10.1186/1471-2180-9-116 PMC2698831 19490622

[54] Sobel JD Ferris D Schwebke J Nyirjesy P Wiesenfeld HC Peipert J Suppressive antibacterial therapy with 0.75% metronidazole vaginal gel to prevent recurrent bacterial vaginosis. Am J Obstet Gynecol. (2006) 194(5):1283–9. 10.1016/j.ajog.2005.11.041 16647911

[55] Srinivasan S Liu C Mitchell CM Fiedler TL Thomas KK Agnew KJ Temporal variability of human vaginal bacteria and relationship with bacterial vaginosis. PLoS One. (2010) 5(4):e10197. 10.1371/journal.pone.0010197 PMC2855365 20419168

[56] Amabebe E Anumba DOC. The vaginal microenvironment: the physiologic role of lactobacilli. Front Med. (2018) 5:181. 10.3389/fmed.2018.00181 PMC6008313 29951482

[57] Abdelmaksoud AA Koparde VN Sheth NU Serrano MG Glascock AL Fettweis JM Comparison of Lactobacillus crispatus isolates from Lactobacillus-dominated vaginal microbiomes with isolates from microbiomes containing bacterial vaginosis-associated bacteria. Microbiology. (2016) 162(3):466–75. 10.1099/mic.0.000238 26747455 PMC4891990

[58] Wu S Hugerth LW Schuppe-Koistinen I Du J. The right bug in the right place: opportunities for bacterial vaginosis treatment. npj Biofilms Microbiomes. (2022) 8(1):1–11. 10.1038/s41522-021-00260-1 35501321 PMC9061781

[59] Mendes-Soares H Suzuki H Hickey RJ Forneya LJ. Comparative functional genomics of Lactobacillus spp. reveals possible mechanisms for specialization of vaginal lactobacilli to their environment. J Bacteriol. (2014) 196(7):1458–70. 10.1128/JB.01439-13 24488312 PMC3993339

[60] Anderson AC Sanunu M Schneider C Clad A Karygianni L Hellwig E Rapid species-level identification of vaginal and oral lactobacilli using MALDI-TOF MS analysis and 16S rDNA sequencing. BMC Microbiol. (2014) 14(1):312. 10.1186/s12866-014-0312-5 25495549 PMC4272787

[61] Ravel J Gajer P Abdo Z Schneider GM Koenig SSK McCulle SL Vaginal microbiome of reproductive-age women. Proc Natl Acad Sci U S A. (2011) 108(Suppl. 1):4680–7. 10.1073/pnas.1002611107 20534435 PMC3063603

[62] Marrazzo JM Fiedler TL Srinivasan S Thomas KK Liu C Ko D Extravaginal reservoirs of vaginal Bacteria as risk factors for incident bacterial vaginosis. J Infect Dis. (2012) 205(10):1580. 10.1093/infdis/jis242 22448002 PMC3415820

[63] Antonio MAD Rabe LK Hillier SL. Colonization of the rectum by Lactobacillus species and decreased risk of bacterial vaginosis. J Infect Dis. (2005) 192(3):394–8. 10.1086/430926 15995952

[64] Senok AC Verstraelen H Temmerman M Botta GA. Probiotics for the treatment of bacterial vaginosis. Cochrane Database Syst Rev. (2009) (4):CD006289. 10.1002/14651858.CD006289.pub2 19821358

[65] van Nood E Vrieze A Nieuwdorp M Fuentes S Zoetendal EG de Vos WM Duodenal infusion of donor feces for recurrent Clostridium difficile. N Engl J Med. (2013) 368(5):407–15. 10.1056/NEJMoa1205037 23323867

[66] Boskey ER Cone RA Whaley KJ Moench TR. Origins of vaginal acidity: high D/l lactate ratio is consistent with bacteria being the primary source. Hum Reprod. (2001) 16(9):1809–13. 10.1093/humrep/16.9.1809 11527880

[67] Wilson J. Managing recurrent bacterial vaginosis. Sex Transm Infect. (2004) 80(1):8–11. 10.1136/sti.2002.002733 14755028 PMC1758381

[68] Dumonceaux TJ Schellenberg J Goleski V Hill JE Jaoko W Kimani J Multiplex detection of bacteria associated with normal microbiota and with bacterial vaginosis in vaginal swabs by use of oligonucleotide-coupled fluorescent microspheres. J Clin Microbiol. (2009) 47(12):4067–77. 10.1128/JCM.00112-09 19794034 PMC2786665

[69] Aldunate M Srbinovski D Hearps AC Latham CF Ramsland PA Gugasyan R Antimicrobial and immune modulatory effects of lactic acid and short chain fatty acids produced by vaginal microbiota associated with eubiosis and bacterial vaginosis. Front Physiol. (2015) 6:164. 10.3389/fphys.2015.00164 26082720 PMC4451362

[70] Holley RL Richter HE Varner RE Pair L Schwebke JR. A randomized, double-blind clinical trial of vaginal acidification versus placebo for the treatment of symptomatic bacterial vaginosis. Sex Transm Dis. (2004) 31(4):236–8. 10.1097/01.OLQ.0000118423.20985.E7 15028938

[71] Boeke AJ Dekker JH Th J Van Eijk M Kostense PJ Dick Bezemer P Effect of lactic acid suppositories compared with oral metronidazole and placebo in bacterial vaginosis: a randomised clinical trial. Genitourin Med. (1993) 69(5):388. 10.1136/sti.69.5.388 8244360 PMC1195125

[72] Fiorilli A Molteni B Milani M. Successful treatment of bacterial vaginosis with a policarbophil-carbopol acidic vaginal gel: results from a randomised double-blind, placebo-controlled trial. Available from: www.elsevier.com/locate/ejogrb (Cited January 14, 2022). 10.1016/j.ejogrb.2004.10.011 15925053

[73] Decena DCD Co JT Manalastas RM Palaypayon EP Padolina CS Sison JM Metronidazole with Lactacyd vaginal gel in bacterial vaginosis. J Obstet Gynaecol Res. (2006) 32(2):243–51. 10.1111/j.1447-0756.2006.00383.x 16594932

[74] Plummer EL Bradshaw CS Doyle M Fairley CK Murray GL Bateson D Lactic acid-containing products for bacterial vaginosis and their impact on the vaginal microbiota: a systematic review. PLoS One. (2021) 16(2):e0246953. 10.1371/journal.pone.0246953 PMC7877752 33571286

[75] Petersen EE Magnani P. Efficacy and safety of Vitamin C vaginal tablets in the treatment of non-specific vaginitis: a randomised, double blind, placebo-controlled study. Eur J Obstet Gynecol Reprod Biol. (2004) 117(1):70–5. 10.1016/j.ejogrb.2004.02.032 15474248

[76] Zodzika J Rezeberga D Donders G Vedmedovska N Vasina O Pundure I Impact of vaginal ascorbic acid on abnormal vaginal microflora. Arch Gynecol Obstet. (2013) 288(5):1039–44. 10.1007/s00404-013-2876-y 23677418

[77] Polatti F Rampino M Magnani P Mascarucci P. Vaginal pH-lowering effect of locally applied vitamin C in subjects with high vaginal pH. Gynecol Endocrinol. (2006) 22(4):230–4. 10.1080/09513590600647441 16723311

[78] Petersen EE Genet M Caserini M Palmieri R. Efficacy of vitamin C vaginal tablets in the treatment of bacterial vaginosis: a randomised, double blind, placebo controlled clinical trial. Arzneimittelforschung. (2011) 61(4):260–5. 10.1055/s-0031-1296197 21650086

[79] Krasnopolsky VN Prilepskaya VN Polatti F Zarochentseva NV Bayramova GR Caserini M Efficacy of vitamin C vaginal tablets as prophylaxis for recurrent bacterial vaginosis: a randomised, double-blind, placebo-controlled clinical trial. J Clin Med Res. (2013) 5(4):309. 10.4021/jocmr1489w 23864922 PMC3712888

[80] Castro J Alves P Sousa C Cereija T França Â Jefferson KK Using an in-vitro biofilm model to assess the virulence potential of bacterial vaginosis or non-bacterial vaginosis Gardnerella vaginalis isolates. Sci Rep. (2015) 5:1–10. 10.1038/srep11640 PMC4481526 26113465

[81] Rosca AS Castro J Sousa LGV Cerca N. Gardnerella and vaginal health: the truth is out there. FEMS Microbiol Rev. (2019) 44(1):73–105. 10.1093/femsre/fuz027 31697363

[82] Tomás M Palmeira-de-Oliveira A Simões S Martinez-de-Oliveira J Palmeira-de-Oliveira R. Bacterial vaginosis: standard treatments and alternative strategies. Int J Pharm. (2020) 587:119659. 10.1016/j.ijpharm.2020.119659 32687973

[83] Swidsinski A Mendling W Loening-Baucke V Swidsinski S Dörffel Y Scholze J An adherent Gardnerella vaginalis biofilm persists on the vaginal epithelium after standard therapy with oral metronidazole. Am J Obstet Gynecol. (2008) 198(1):97.e1–6. 10.1016/j.ajog.2007.06.039 18005928

[84] Swidsinski A Dörffel Y Loening-Baucke V Schilling J Mendling W. Response of Gardnerella vaginalis biofilm to 5 days of moxifloxacin treatment. FEMS Immunol Med Microbiol. (2011) 61(1):41–6. 10.1111/j.1574-695X.2010.00743.x 20955467

[85] Muzny CA Schwebke JR. Biofilms: an underappreciated mechanism of treatment failure and recurrence in vaginal infections. Clin Infect Dis. (2015) 61(4):601–6. 10.1093/cid/civ353 25935553 PMC4607736

[86] Machado D Castro J Palmeira-de-Oliveira A Martinez-de-Oliveira J Cerca N. Bacterial vaginosis biofilms: challenges to current therapies and emerging solutions. Front Microbiol. (2016) 6:1528. 10.3389/fmicb.2015.01528 26834706 PMC4718981

[87] Machado D Gaspar C Palmeira-De-Oliveira A Cavaleiro C Salgueiro L Martinez-De-Oliveira J Thymbra capitata essential oil as potential therapeutic agent against Gardnerella vaginalis biofilm-related infections. Future Microbiol. (2017) 12(5):407–16. 10.2217/fmb-2016-0184 28339292

[88] Rosca AS Castro J Sousa LGV França A Cavaleiro C Salgueiro L Six bacterial vaginosis-associated species can form an in vitro and ex vivo polymicrobial biofilm that is susceptible to Thymbra capitata essential oil. Front Cell Infect Microbiol. (2022) 12:1. 10.3389/fcimb.2022.824860 PMC9114774 35601098

[89] Mendling W Weissenbacher ER Gerber S Prasauskas V Grob P. Use of locally delivered dequalinium chloride in the treatment of vaginal infections: a review. Arch Gynecol Obstet. (2016) 293(3):469–84. 10.1007/s00404-015-3914-8 26506926 PMC4757629

[90] Della Casa V Noll H Gonser S Grob P Graf F Pohlig G. Antimicrobial activity of dequalinium chloride against leading germs of vaginal infections. Arzneimittel-Forschung/Drug Res. (2002) 52(9):699–705. 10.1055/s-0031-1299954 12404886

[91] Gaspar C Rolo J Cerca N Palmeira-De-oliveira R Martinez-De-oliveira J Palmeira-De-oliveira A. Dequalinium chloride effectively disrupts bacterial vaginosis (BV) Gardnerella spp. Biofilms. Pathogens. (2021) 10(3):1–11. 10.3390/pathogens10030261 PMC7996269 33668706

[92] Weissenbacher ER Donders G Unzeitig V Martinez De Tejada B Gerber S Halaška M A comparison of dequalinium chloride vaginal tablets (Fluomizin®) and clindamycin vaginal cream in the treatment of bacterial vaginosis: a single-blind, randomized clinical trial of efficacy and safety. Gynecol Obstet Invest. (2012) 73(1):8–15. 10.1159/000332398 22205034

[93] Landlinger C Oberbauer V Tisakova LP Schwebs T Berdaguer R Van Simaey L Preclinical data on the gardnerella-specific endolysin PM-477 indicate its potential to improve the treatment of bacterial vaginosis through enhanced biofilm removal and avoidance of resistance. Antimicrob Agents Chemother. (2022) 66(5):e0231921. 10.1128/aac.02319-21 35416708 PMC9112913

[94] Castro J Sousa LGV França Â Tisakova LP Corsini L Cerca N. Exploiting the Anti-Biofilm Effect of the Engineered Phage Endolysin PM-477 to Disrupt In Vitro Single- and Dual-Species Biofilms of Vaginal Pathogens Associated with Bacterial Vaginosis. (2022). 10.3390/antibiotics11050558 PMC9137943 35625202

[95] Youn CK Jun Y Jo ER Jang SJ Song H Cho SI. Comparative efficacies of topical antiseptic eardrops against biofilms from methicillin-resistant Staphylococcus aureus and quinolone-resistant Pseudomonas aeruginosa. J Laryngol Otol. (2018) 132(6):519–22. 10.1017/S0022215118000932 29909794

[96] Grønseth T Vestby LK Nesse LL Thoen E Habimana O von Unge M Lugol’s solution eradicates Staphylococcus aureus biofilm in vitro. Int J Pediatr Otorhinolaryngol. (2017) 103:58–64. 10.1016/j.ijporl.2017.09.025 29224767

[97] Powell A Ghanem KG Rogers L Zinalabedini A Brotman RM Zenilman J Clinicians’ use of intravaginal boric acid maintenance therapy for recurrent vulvovaginal candidiasis and bacterial vaginosis. Sex Transm Dis. (2019) 46(12):810. 10.1097/OLQ.0000000000001063 31663976 PMC6878170

[98] Surapaneni S Akins R Sobel JD. Recurrent bacterial vaginosis: an unmet therapeutic challenge. Experience with a combination pharmacotherapy long-term suppressive regimen. Sex Transm Dis. (2021) 48(10):761. 10.1097/OLQ.0000000000001420 34110746 PMC8460079

[99] Marrazzo JM Dombrowski JC Wierzbicki MR Perlowski C Pontius A Dithmer D Safety and efficacy of a novel vaginal anti-infective, TOL-463, in the treatment of bacterial vaginosis and vulvovaginal candidiasis: a randomized, single-blind, phase 2, controlled trial. Clin Infect Dis. (2019) 68(5):803–9. 10.1093/cid/ciy554 30184181 PMC6376090

[100] Fehir R. Chemicals evaluated for carcinogenic potential by the office of pesticide programs. Washington, DC (2021) http://npic.orst.edu/chemicals_evaluated.pdf.

[101] Waldbaum AS Schwebke JR Paull JRA Price CF Edmondson SR Castellarnau A A phase 2, double-blind, multicenter, randomized, placebo-controlled, dose-ranging study of the efficacy and safety of Astodrimer Gel for the treatment of bacterial vaginosis. PLoS One. (2020) 15(5):e0232394. 10.1371/journal.pone.0232394 32365097 PMC7197797

[102] Chavoustie SE Carter BA Waldbaum AS Donders GGG Peters KH Schwebke JR Two phase 3, double-blind, placebo-controlled studies of the efficacy and safety of Astodrimer 1% Gel for the treatment of bacterial vaginosis. Eur J Obstet Gynecol Reprod Biol. (2020) 245:13–8. 10.1016/j.ejogrb.2019.11.032 31812702

[103] Arcavi L Benowitz NL. Cigarette smoking and infection. Arch Intern Med. (2004) 164(20):2206–16. 10.1001/archinte.164.20.2206 15534156

[104] Nelson DB Bellamy S Odibo A Nachamkin I Ness RB Allen-Taylor L. Vaginal symptoms and bacterial vaginosis (BV): how useful is self-report? Development of a screening tool for predicting BV status. Epidemiol Infect. (2007) 135(8):1369. 10.1017/S095026880700787X 17274857 PMC2870698

[105] Cherpes TL Hillier SL Meyn LA Busch JL Krohn MA. A delicate balance: risk factors for acquisition of bacterial vaginosis include sexual activity, absence of hydrogen peroxide-producing lactobacilli, black race, and positive herpes simplex virus type 2 serology. Sex Transm Dis. (2008) 35(1):78–83. 10.1097/OLQ.0b013e318156a5d0 17989585

[106] Brotman RM He X Gajer P Fadrosh D Sharma E Mongodin EF Association between cigarette smoking and the vaginal microbiota: a pilot study. BMC Infect Dis. (2014) 14:471. 10.1186/1471-2334-14-471 25169082 PMC4161850

[107] Nelson TM Borgogna JC Michalek RD Roberts DW Rath JM Glover ED Cigarette smoking is associated with an altered vaginal tract metabolomic profile. Sci Rep.. (2018) 8(1):1–13. 10.1038/s41598-017-14943-3 29339821 PMC5770521

[108] Faleiro L Miguel G Gomes S Costa L Venâncio F Teixeira A Antibacterial and antioxidant activities of essential oils isolated from Thymbra capitata L. (Cav.) and Origanum vulgare L. J Agric Food Chem. (2005) 53(21):8162–8. 10.1021/jf0510079 16218659

[109] Eleuterio J Giraldo PC Silveira Gonçalves AK Nunes Eleuterio RM. Liquid-based cervical cytology and microbiological analyses in women using cooper intrauterine device and levonorgestrel-releasing intrauterine system. Eur J Obstet Gynecol Reprod Biol. (2020) 255:20–4. 10.1016/j.ejogrb.2020.09.051 33065517

[110] Peebles K Kiweewa FM Palanee-Phillips T Chappell C Singh D Bunge KE Elevated risk of bacterial vaginosis among users of the copper intrauterine device: a prospective longitudinal cohort study. Clin Infect Dis. (2021) 73(3):513–20. 10.1093/cid/ciaa703 32505132 PMC8326546

[111] Hutchinson KB Kip KE Ness RB. Condom use and its association with bacterial vaginosis and bacterial vaginosis-associated vaginal microflora. Epidemiology. (2007) 18(6):702–8. 10.1097/EDE.0b013e3181567eaa 17917605

[112] Shivakoti R Tuddenham S Caulfield LE Murphy C Robinson C Ravel J Dietary macronutrient intake and molecular-bacterial vaginosis: role of fiber. Clin Nutr. (2020) 39(10):3066–71. 10.1016/j.clnu.2020.01.011 32033845 PMC7387193

[113] Turner AN Carr Reese P Fields KS Anderson J Ervin M Davis JA A blinded, randomized controlled trial of high-dose vitamin D supplementation to reduce recurrence of bacterial vaginosis. Am J Obstet Gynecol. (2014) 211(5):479.e1–479.e13. 10.1016/j.ajog.2014.06.023 24949544 PMC4254061

[114] Mohammadzadeh F Dolatian M Jorjani M Majd HA Borumandnia N. Comparing the therapeutic effects of garlic tablet and oral metronidazole on bacterial vaginosis: a randomized controlled clinical trial. Iran Red Crescent Med J. (2014) 16(7):e19118. 10.5812/ircmj.19118 PMC4166107 25237588

[115] Tuddenham S Stennett CA Cone RA Ravel J Macintyre AN Ghanem KG Vaginal cytokine profile and microbiota before and after lubricant use compared with condomless vaginal sex: a preliminary observational study. BMC Infect Dis. (2021) 21(1):1–13. 10.1186/s12879-021-06512-x 34537015 PMC8449901

[116] Hammer KA Carson CF Riley TV. In vitro susceptibilities of lactobacilli and organisms associated with bacterial vaginosis to Melaleuca alternifolia (tea tree) oil. Antimicrob Agents Chemother. (1999) 43(1):196. 10.1128/AAC.43.1.196 10094671 PMC89050

[117] Ma X Deng J Cui X Chen Q Wang W. Berberine exhibits antioxidative effects and reduces apoptosis of the vaginal epithelium in bacterial vaginosis. Exp Ther Med. (2019) 18(3):2122–30. 10.3892/etm.2019.7772 PMC6676195 31410167

[118] Ness RB Hillier SL Richter HE Soper DE Stamm C McGregor J Douching in relation to bacterial vaginosis, lactobacilli, and facultative bacteria in the vagina. Obstet Gynecol. (2002) 100(4):765–72. 10.1016/s0029-7844(02)02184-1 12383547

[119] Marconi C El-Zein M Ravel J Ma B Lima MD Carvalho NS Characterization of the vaginal microbiome in women of reproductive age from 5 Regions in Brazil. Sex Transm Dis. (2020) 47(8):562–9. 10.1097/OLQ.0000000000001204 32520883

[120] Lokken EM Manguro GO Abdallah A Ngacha C Shafi J Kiarie J Association between vaginal washing and detection ofLactobacillus by culture and quantitative PCR inHIV-seronegative Kenyan women: a cross-sectional analysis. Sex Transm Infect. (2019) 95(6):455. 10.1136/sextrans-2018-053769 30696752 PMC7053826

[121] Sabo MC Balkus JE Richardson BA Srinivasan S Kimani J Anzala O Association between vaginal washing and vaginal bacterial concentrations. PLoS One. (2019) 14(1):e0210825. 10.1371/journal.pone.0210825 PMC6345501 30677048

[122] Tamarelle J Shardell MD Ravel J Brotman RM. Factors associated with incidence and spontaneous clearance of molecular-bacterial vaginosis: results from a longitudinal frequent-sampling observational study. Sex Transm Dis. (2022) 49(9):649–56. 10.1097/OLQ.0000000000001662 35969846 PMC9387550

[123] Brotman RM Ghanem KG Klebanoff MA Taha TE Scharfstein DO Zenilman JM. The effect of vaginal douching cessation on bacterial vaginosis: a pilot study. Am J Obstet Gynecol. (2008) 198(6):628.e1–e7. 10.1016/j.ajog.2007.11.043 18295180 PMC2494605

[124] Tuddenham S Ghanem KG Caulfield LE Rovner AJ Robinson C Shivakoti R Associations between dietary micronutrient intake and molecular-bacterial vaginosis. Reprod Health. (2019) 16(1):151. 10.1186/s12978-019-0814-6 31640725 PMC6806504

[125] Yamada Y Azuma K. Evaluation of the in vitro antifungal activity of allicin. Antimicrob Agents Chemother. (1977) 11(4):743. 10.1128/AAC.11.4.743 856026 PMC352060

[126] Akoh CC Pressman EK Cooper E Queenan RA Pillittere J O’Brien KO. Low vitamin D is associated with infections and proinflammatory cytokines during pregnancy. Reprod Sci. (2018) 25(3):414–23. 10.1177/1933719117715124 28618852 PMC6343221

[127] Bodnar LM Krohn MA Simhan HN. Maternal vitamin D deficiency is associated with bacterial vaginosis in the first trimester of pregnancy. J Nutr. (2009) 139(6):1157–61. 10.3945/jn.108.103168 19357214 PMC2682987

[128] Dunlop AL Taylor RN Tangpricha V Fortunato S Menon R. Maternal vitamin D, folate, and polyunsaturated fatty acid status and bacterial vaginosis during pregnancy. Infect Dis Obstet Gynecol. (2011) 2011:216217. 10.1155/2011/216217 22190843 PMC3235789

[129] Klebanoff MA Turner AN. Bacterial vaginosis and season, a proxy for vitamin D status. Sex Transm Dis. (2014) 41(5):295–9. 10.1097/OLQ.0000000000000124 24722382 PMC4267683

[130] Rafat D Singh S Nawab T Khan F Khan AU Khalid S. Association of vaginal dysbiosis and gestational diabetes mellitus with adverse perinatal outcomes. Int J Gynecol Obstet. (2022) 158(1):70–8. 10.1002/ijgo.13945 34561861

[131] Donders GGG. Lower genital tract infections in diabetic women. Curr Infect Dis Rep. (2002) 4(6):536–9. 10.1007/s11908-002-0042-y 12433331

[132] Yagnik D Serafin V Shah AJ. Antimicrobial activity of apple cider vinegar against Escherichia coli, Staphylococcus aureus and Candida albicans; downregulating cytokine and microbial protein expression. Sci Rep. (2018) 8(1):1–12. 10.1038/s41598-017-18618-x 29379012 PMC5788933

[133] Dezzutti CS Brown ER Moncla B Russo J Cost M Wang L Is wetter better? An evaluation of over-the-counter personal lubricants for safety and anti-HIV-1 activity. PLoS One. (2012) 7(11):e48328. 10.1371/journal.pone.0048328 23144863 PMC3492332

[134] Adriaens E Remon JP. Mucosal irritation potential of personal lubricants relates to product osmolality as detected by the slug mucosal irritation assay. Sex Transm Dis. (2008) 35(5):512–6. 10.1097/OLQ.0b013e3181644669 18356773

[135] World Health Organization Use and procurement of additional lubricants for male and female condoms: WHO/UNFPA/FHI360: advisory note. World Health Organization. (2012). https://apps.who.int/iris/handle/10665/76580.

[136] Hung KJ Hudson PL Bergerat A Hesham H Choksi N Mitchell C. Effect of commercial vaginal products on the growth of uropathogenic and commensal vaginal bacteria. Sci Rep.. (2020) 10(1):1–6. 10.1038/s41598-021-02426-5 32376907 PMC7203152

[137] Wilkinson EM Łaniewski P Herbst-Kralovetz MM Brotman RM. Personal and clinical vaginal lubricants: impact on local vaginal microenvironment and implications for epithelial cell host response and barrier function. J Infect Dis. (2019) 220(12):2009–18. 10.1093/infdis/jiz412 31539059 PMC6834067

[138] Smith-McCune K Chen JC Greenblatt RM Shanmugasundaram U Shacklett BL Hilton JF Unexpected inflammatory effects of intravaginal gels (universal placebo gel and nonoxynol-9) on the upper female reproductive tract: a randomized crossover study. PLoS One. (2015) 10(7):e0129769. 10.1371/journal.pone.0129769 26177352 PMC4503751

[139] Edwards D Panay N. Treating vulvovaginal atrophy/genitourinary syndrome of menopause: how important is vaginal lubricant and moisturizer composition? Climacteric. (2016) 19(2):151–61. 10.3109/13697137.2015.1124259 26707589 PMC4819835

[140] Hesham H Mitchell AJ Bergerat A Hung K Mitchell CM. Impact of vaginal douching products on vaginal Lactobacillus, Escherichia coli and epithelial immune responses. Sci Reports 2021 111. (2021) 11(1):1–8. 10.1038/s41598-021-02426-5 PMC8629978 34845288

[141] Turpin R Tuddenham S He X Klebanoff MA Ghanem KG Brotman RM. Bacterial vaginosis and behavioral factors associated with incident pelvic inflammatory disease in the longitudinal study of vaginal Flora. J Infect Dis. (2021) 224(12 Suppl 2):S137–44. 10.1093/infdis/jiab103 34396403 PMC8499701

[142] Zhang R Zhu X Bai H Ning K. Network pharmacology databases for traditional Chinese medicine: review and assessment. Front Pharmacol. (2019) 10:123. 10.3389/fphar.2019.00123 PMC6393382 30846939

[143] Zhao H Zhao L Wu F Shen L. Clinical research on traditional Chinese medicine treatment for bacterial vaginosis. Phyther Res. (2021) 35(9):4943–56. 10.1002/ptr.7123 33860974

[144] Blackwell AL. Tea tree oil and anaerobic (bacterial) vaginosis. Lancet. (1991) 337(8736):300. 10.1016/0140-6736(91)90910-H 1671134

[145] Delgado-Adámez J Garrido M Bote ME Fuentes-Pérez MC Espino J Martín-Vertedor D. Chemical composition and bioactivity of essential oils from flower and fruit of Thymbra capitata and Thymus species. J Food Sci Technol. (2017) 54(7):1857. 10.1007/s13197-017-2617-5 28720941 PMC5495709

[146] Karampoula F Giaouris E Deschamps J Doulgeraki AI Nychas GJE Dubois-Brissonnet F. Hydrosol of thymbra capitata is a highly efficient biocide against Salmonella enterica serovar Typhimurium biofilms. Appl Environ Microbiol. (2016) 82(17):5309. 10.1128/AEM.01351-16 27342550 PMC4988213

