# Supplementary material for: Bacterial vaginosis: a review of approaches to treatment and prevention
Source: Front Reprod Health. 2023 May 31;5:1100029. doi: 10.3389/frph.2023.1100029 (PMC10264601; doi:10.3389/frph.2023.1100029)
Supplement: Supplementary file 1 [file Table1.docx]

**Supplemental Table 1.** Methodology on assessment and appraisal of literature. topic

| **Literature Appraisal Methodology** |
| --- |
| - In assessing research studies included in this paper, the following were considered: Population size, type of study, risk of bias, precision, consistency, directness - Discussion of these variables are included in the text body as applicable - Quality of evidence was qualified by GRADE (Grading of Recommendations, Assessment, Development and Evaluations) certainty ratings, which outline a systematic approach for authors to appraise literature (144). GRADE ratings were used to appraise beneficial effect on the management and/or prevention of bacterial vaginosis   GRADE ratings used include:  Low (true effect may be different from estimated effect)  Moderate (true effect is likely close to estimated effect)  High (confidence that true effect is similar to estimated effect)   - In fields with numerous research studies with low to moderate certainty ratings, studies were filtered by population (N >80). For example, the section on probiotics. - In fields with limited research with low to moderate certainty ratings, all population sizes were included to allow for complete appraisal of where the field stands. For example, the section on vaginal microbiota transplant. |
